# African AIDS Vaccine Programme for a Coordinated and Collaborative Vaccine Development Effort on the Continent

**DOI:** 10.1371/journal.pmed.0050236

**Published:** 2008-12-02

**Authors:** Pontiano Kaleebu, Alash'le Abimiku, Shenaaz El-Halabi, Sinata Koulla-Shiro, Nicole Mamotte, Souleymane Mboup, Roy Mugerwa, John Nkengasong, Coumba Toure-Kane, Tim Tucker, Douglas Wassenaar, Carolyn Williamson, Dawit Wolday

## Abstract

The African AIDS Vaccine Programme, formed in 2000, is a network of African HIV vaccine stakeholders, led by Africans across the continent, with a vision of an African continent without AIDS.

Nearly 30 years after the first cases of AIDS were reported [1] and HIV identified as the causative agent, Africa today bears a disproportionate burden of the disease. Sub-Saharan Africa has just over 10% of the world's population but is home to more than 65% of the 33 million people in the world estimated to be living with HIV/AIDS. The annual rate of new infections continues to rise, with an estimated 2.5 million people newly infected in 2007 [2].

Although newer initiatives aimed at providing universal access to antiretroviral therapy in Africa are ongoing, most Africans receive neither treatment nor adequate care [3]. Relatively few HIV-infected patients in sub-Saharan Africa (about 1 million) are currently receiving antiretroviral treatment [4], and many more new cases are occurring each year [2]. It is thus evident that treatment alone will not control the spread of the disease and that a vaccine is our best hope of halting this pandemic.

In the history of infectious diseases, effective vaccines are critical public health tools to control and prevent diseases. Developing an effective HIV vaccine is proving to be one of the greatest challenges in biomedical research. The need for a simple, safe, effective, affordable, and equitably distributed preventive HIV vaccine to complement existing HIV/AIDS prevention, treatment, and care programs is greater than ever. It is probable that vaccines will form part of future innovative interventions including microbicides and circumcision, used in conjunction with other more traditional prevention interventions.

**Summary Points**

- The AIDS pandemic continues to be the most serious public health challenge facing the world today, and Africa bears an overwhelming proportion of infections, with unprecedented medical and socioeconomic consequences.
- The best hope to end the AIDS pandemic remains the development of an effective HIV vaccine, and its distribution to all communities.
- The African AIDS Vaccine Programme (AAVP), formed in 2000, is a network of African HIV vaccine stakeholders, led by Africans across the continent, with a vision of an African continent without AIDS.
- AAVP supports and represents the diverse African communities involved in HIV vaccine research and development (R&D), and is an important unified voice for African stakeholders.
- This paper describes what AAVP is, what it does, and its impact, successes, and challenges. Finally, we discuss where AAVP is heading to harmonize with the Global HIV Vaccine Enterprise and the dynamic HIV vaccine research field.

## What Is AAVP?

### An African response—the genesis of AAVP.

AAVP was created in 2000 within the global context of relatively little attention being paid to Africa's need for an HIV vaccine. While an HIV vaccine has always been considered a global public good, most laboratory and clinical activities prior to 2000 were conducted in the industrialized world with candidate vaccines that were not based on strains circulating in Africa. AAVP was created with the specific intention of mobilizing support and advocating for a more Africa-focused vaccine agenda. In 2000, representative African scientists met in Nairobi to create AAVP and issued a declaration—“An African Appeal for an AIDS Vaccine” [5].

The mission of AAVP is to advocate for and support a coordinated international effort to promote the development of and future access to HIV vaccines suitable for use in Africa. To meet the vision of an AIDS-free Africa through an effective vaccine, AAVP has strengthened the network of African HIV vaccine stakeholders through research, advocacy, partnership, and contribution to capacity strengthening and policy development. AAVP aims to ensure that African communities and Africans participating in the HIV vaccine development process are adequately supported, are well represented globally, and are equal partners playing a critical role in HIV vaccine development efforts. AAVP is supported by the World Health Organization (WHO) and the Joint United Nations Programme on HIV/AIDS (UNAIDS). AAVP is a partnership-based network, committed to working closely with all HIV vaccine partners and sharing its resources and capacity, consistent with the Global HIV Vaccine Enterprise. AAVP has been successful in raising funds from different sectors and countries for its activities since its inception.

AAVP (Figure 1) is coordinated by a secretariat and a steering committee (SC). The secretariat is housed by the WHO Initiative for Vaccine Research and its HIV Vaccine Initiative, with plans for relocation to an African center in the future. This future secretariat will maintain close ties to WHO, but will also have strong collaborations and links to the Global HIV Vaccine Enterprise activities and planned secretariat in Africa. The AAVP SC is an advisory body comprised of senior African stakeholders in the field of HIV vaccines. The SC advises the secretariat on unique opportunities for collaboration, and provides strategic directions for AAVP. The SC also has representation from the international partners to facilitate networking, promote transparency, and consolidate global involvement.

**Figure 1:**
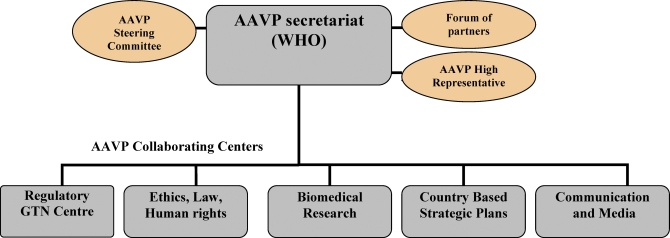
The AAVP Structure GTN, Global Training Network.

AAVP operates and implements most of its activities through a network of collaborative centers (CCs), located at key institutions across the continent, selected for their extensive experience and comparative advantages to AAVP and their ability for technology transfer within the continent. There are five CCs, which reflect AAVP's primary work areas: (1) regulatory issues; (2) ethics, law, and human rights; (3) biomedical research; (4) country-based strategic planning; and (5) communication and media. Table 1 shows their location and key functions. All CCs are guided by the following strategic directions: capacity strengthening, advocacy, policy development, increased community involvement, and networking (Figure 2).

**Table 1:**
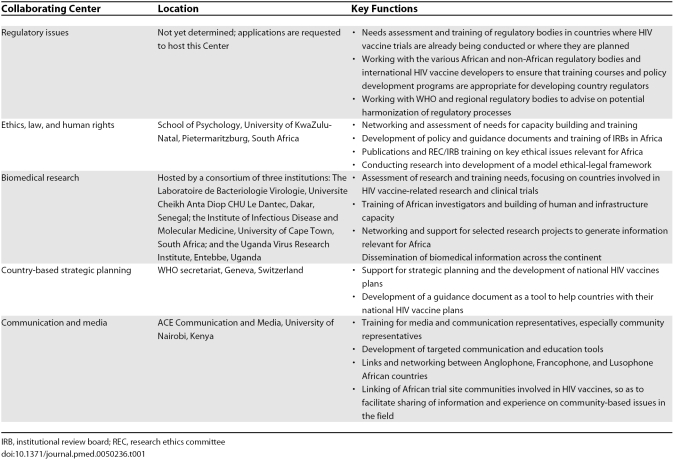
AAVP Collaborating Centers, Location, and Key Functions

**Figure 2:**
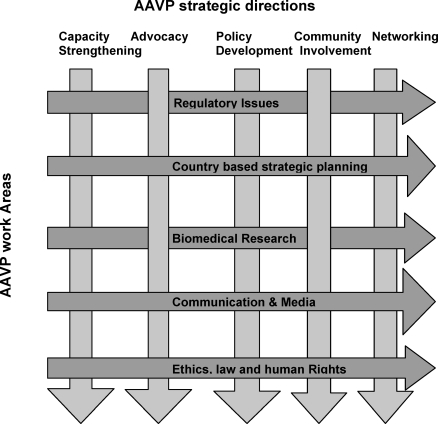
AAVP Strategic Directions and Work Areas

To ensure AAVP is adequately represented at a political level, Madame Jeannette Kagame, First Lady of Rwanda, has been appointed as AAVP's “High Representative.” She is well versed in the issues of poverty-associated diseases, and has a high profile within Africa. The High Representative is expected to drive the program at a political level and act as AAVP's “Ambassador” at various meetings and policy forums. The AAVP High Representative will work closely with the AAVP secretariat, the AAVP CCs, the secretariat of the Global HIV Vaccine Enterprise, and the relevant WHO structures that are set up to influence and advocate for countries—e.g., the WHO External Relations Officer and the New Partnership for African Development (NEPAD; http://www.nepad.org/) Focal Point.

## What Does AAVP Do?

As a network of African stakeholders in vaccine development, AAVP has the comparative advantage of being in a unique position to provide common African solutions across the diverse cultural and political environment on the continent. Africa's participation must include all aspects of vaccine development: laboratory research, vaccine and trial design, cohort development, advocacy, ethical, and regulatory issues, and projections and strategies for use of any possible HIV vaccine. AAVP also has the advantage of impartiality in that it has no specific products in development itself; AAVP's close linkage to WHO/UNAIDS with their experience in ethics, regulatory, and policy issues gives it greater leverage in this regard.

Operating through the five CCs, AAVP addresses identified gaps, such as the lack of adequate global regulatory capacity and harmonization, which is a major impediment to the conduct of properly reviewed and monitored, high-quality HIV vaccine clinical trials. AAVP also recognizes that the development of an HIV vaccine requires significant attention to ethics, law, and human rights, and works at the interface of these fields with behavioral sciences to address the key issues in HIV vaccine R&D and trials.

Furthermore, the AAVP vision requires that Africans be integral to all aspects of vaccine development. Increased African participation in HIV vaccine-related biomedical research is critical for true partnership and trust. AAVP strengthens and develops a broad base of African biomedical scientists and research clinicians, who are able to respond to relevant research questions and have the skills to secure further national and international funding in collaboration with their counterparts in the developed world. AAVP also assists countries with the development of country-specific vaccine R&D plans and ensures that these are integrated as part of the overall national AIDS control program of each country. Finally, AAVP focuses on increasing the capacity of stakeholders on the continent to communicate more effectively on HIV vaccines.

## Specific Projects, Impact, and Successes

### AAVP achievements.

Since its inception, AAVP has contributed significantly to a new level of visibility for African HIV vaccine activities and has played an important coordinating and networking role on the continent. AAVP has brought together various African HIV vaccine stakeholders from many countries, creating collaborations and training programs and organizing symposia addressing key issues relevant to Africa. One year after its formation, in the 2001 Abuja Declaration on HIV/AIDS, Tuberculosis, and Malaria, the African Union Heads of State and Government committed themselves to supporting the development of an effective, affordable, accessible HIV vaccine relevant to Africa. They also pledged to support AAVP and its partners and institutions devoted to HIV vaccine research and testing in Africa. This event, similar to the endorsement of the Global HIV Vaccine Enterprise by the G8 [6], is significant, as it was the first time African leaders collectively and strongly endorsed a continent-based vaccine effort.

To date, AAVP has successfully expanded and increased training and capacity building in the areas of community and media education, ethics, laboratory, and good clinical and regulatory practices specific to HIV vaccine development. AAVP has also provided support to the development of national AIDS vaccine plans in Botswana, Cameroon, Côte d'Ivoire, Ethiopia, Kenya, Nigeria, Rwanda, Tanzania, Uganda, and Zambia, and to the involvement of community groups in preparation for HIV vaccine trials. AAVP has been instrumental in the development of regional networks and collaborative partnerships with African HIV vaccine research stakeholders. It has facilitated an international discussion of the inclusion of adolescents in HIV vaccine trials [7], identified capacity needs of 35 African research ethics committees to review HIV vaccine trials [8], and developed discussion documents on topics such as enrollment of women in trials [9], intellectual property, and tissue transfer issues [10]. AAVP has provided technical and financial support to ten research projects implemented by young African scientists (e.g., [11]). In addition, AAVP has organized three forums to facilitate interaction between partners and stakeholders for the development of common policies and strategies and their integration into the global vaccine agenda.

## Challenges

### Changes in the global HIV vaccine environment.

Since the creation of AAVP in 2000, there have been dramatic changes in the African and global HIV vaccine context. The HIV vaccine R&D agenda has become far more focused on countries most affected by the HIV pandemic, and investment in Africa has increased significantly. In addition, there has been a shift by vaccine developers towards vaccine candidates that better reflect the HIV-1 variants prevalent in Africa. One indicator of the increased focus on Africa can be seen in the number of countries conducting HIV vaccine trials. Only one country (Uganda) had conducted an HIV vaccine clinical trial prior to 2000 [12], whereas by 2007, eight African countries had conducted HIV vaccine trials (http://www.iavireport.org/) (Figure 3). In addition, the level of political support has also increased in many African countries.

**Figure 3:**
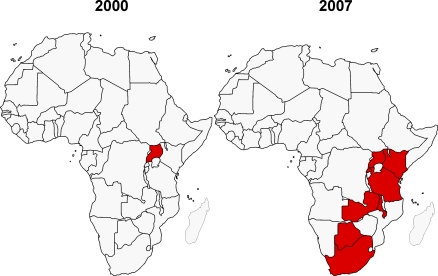
African Countries Participating in HIV Vaccine Trials

Global changes in the field of HIV vaccines, to which AAVP has responded, have occurred within two major contexts. First, there has been a growing understanding by the international community of the need to work more closely with scientists, clinicians, communities, politicians, and other stakeholders in highly affected regions of the globe, as well as to work with more relevant vaccine technology platforms and HIV gene inserts. The move to shift greater financial support to developing countries has required increased capacity development and political support within developed countries.

Second, the significant shift in global thinking associated with the Global HIV Vaccine Enterprise has changed the field. The Enterprise is an alliance of independent global HIV vaccine development partners, all of which have their own work plans, but share a commitment to working together in a more efficient, collaborative, and complementary manner to develop an HIV vaccine in the shortest possible time (http://www.hivvaccineenterprise.org/) [13].

## Where Is AAVP Heading?

In the next two to three years, AAVP will become an autonomous organization, with its secretariat based in Africa but with strong links to WHO/UNAIDS and the Global HIV Vaccine Enterprise. This move will bring AAVP closer to its partners working in Africa and to African leadership, and will increase African ownership. The CCs will be central to AAVP's activities, acting as hubs bringing together partners to address a shared plan aligned with the Global HIV Vaccine scientific plan. While biomedical research, communication and media, ethics, law, and human rights, and regulatory issues will be based at the CCs with coordination at the secretariat, country-based strategic planning will remain an activity of the secretariat working closely with WHO/UNAIDS. An important strategic objective for AAVP will be to influence the global agenda for HIV vaccine R&D and, at the same time, to create stronger ties and partnerships between African researchers and institutions and the rest of the world. To that end, AAVP will explore the possibility of a close relationship with the Global HIV Vaccine Enterprise as a vehicle for positioning African HIV vaccine research internationally.

In summary, AAVP acts to serve the network of African stakeholders in the major global mobilization to discover an effective HIV vaccine. AAVP is entering a new phase of increased activities and exposure, subsequent to comprehensive external review and strategic planning processes.

AAVP is firmly positioned to play a significant leadership role on the African continent as the voice of African stakeholders involved in HIV vaccine development. AAVP's plans are responding to the changing HIV vaccine environment, and offer substantial added value to those working in Africa by focusing on areas where AAVP has a comparative advantage. AAVP looks forward to the next five years of enhanced activities and greater impact towards its vision of “an AIDS-free Africa through an effective vaccine.”

## Abbreviations

AAVP: African AIDS Vaccine Programme
CC: collaborative center
R&D: research and development
SC: steering committee
UNAIDS: Joint United Nations Programme on HIV/AIDS
WHO: World Health Organization
